# Biomarkers in SIRS and sepsis: Quo vadis?

**DOI:** 10.4103/0974-2700.58665

**Published:** 2010

**Authors:** Alexander R. Novotny

**Affiliations:** Department of Surgery, Klinikum rechts der Isar, Technische Universitaet Muenchen, Munich, Germany

Systemic inflammatory response syndrome (SIRS) and sepsis are still a major cause of in-hospital morbidity and mortality.[1] SIRS and sepsis are diagnosed clinically according to the American College of Chest Physicians/Society of Critical Care Medicine (ACCP/SCCM) criteria.[2] Hereby, a SIRS with proven infection is referred to as sepsis. However, in clinical routine, it is often difficult to isolate the microbial inoculum, making the differential diagnosis between SIRS and sepsis difficult. This differential diagnosis is in turn crucial for further therapeutic decisions: is an antimicrobial therapy and aggressive search for a septic focus with all its side-effects necessary or is a focussed symptomatic therapy of the SIRS the adequate treatment concept? Therefore, the concept of using biomarkers to differentiate SIRS from sepsis is appealing.[3,4]

Apart from their potential in discriminating SIRS from sepsis, numerous biochemical indicators have been evaluated regarding their potential in predicting sepsis prognosis.[5] In the setting of postoperative sepsis for example, these can be divided into markers for pre-operative risk evaluation, markers for peri-operative risk evaluation and those for an early prediction of sepsis prognosis. Markers for pre-operative risk assessment of sepsis-related death are the interleukin (IL)-12 production after either lipopolysaccharide (LPS)stimulation of isolated monocytes[6] or whole blood.[7] It might be hypothesized that the capacity of monocytes to synthesize IL-12 may also be used as a tool for risk assessment in other types of sepsis than septic peritonitis. Markers for peri-operative risk evaluation, which assess the constitutional risk of sepsis-related death, include the tumor necrosis factor (TNF)-β gene polymorphism,[8] the TNF promotor polymorphism,[9] the IL-1ra polymorphism and the IL-1 gene polymorphism.[10] Biomarkers for early prediction of sepsis prognosis include procalcitonin (PCT),[11] IL-6,[12] IL-18[13] and NF-xB activity.[14]

Hitherto, no single biomarker for prognosis prediction found widespread entrance into clinical routine. In most publications, a combination of biomarkers with clinical scoring systems,[11] like the APACHE 2 score, yielded the highest specificity and sensitivity in predicting patient prognosis.

These days, the most useful biomarker for routine clinical use seems to be PCT due to its high specificity for bacterial and fungal infections and its adequately sized diagnostic window due to a half-life of approximately 20–24 h. In the setting of postoperative sepsis, it is of special advantage that PCT levels are only slightly influenced by the operative trauma itself.[15]

With a more thorough understanding of the underlying immune responses in sepsis, the number of potential markers for prognosis prediction is steadily growing.

This is the context in which the manuscript “A Biomarker Panel to Discriminate between SIRS and Sepsis and Sepsis Severity” has to be seen. The authors provide evidence that metalloproteinases and sE-selectin are differentially regulated in SIRS and sepsis and can therefore be of use to distinguish between these two forms. Furthermore, they were able to identify a set of biomarkers predicting patient outcome with high specificity and sensitivity. As the authors conclude, these results should give rise to a validation study in a larger and preferably more homogenous set of patients.

Forthcoming investigations dealing with prognosis prediction in sepsis will most probably be based on microarray technology, making the investigation of a large number of potential prognosticators possible at the same time. For an effective use of this method, however, you have to know what you are looking for; otherwise, this method is comparable to dynamite fishing. Therefore, investigations providing insight into the immunologic mechanisms in SIRS and sepsis *in vivo* are of crucial importance. Accordingly, the set of “usual suspects” for prospective research in this field should be extended to the biomarkers proposed in “A Biomarker Panel to Discriminate between SIRS and Sepsis and Sepsis Severity.”

Apart from biomarkers enabling prognostic drawbacks, one might hit upon regulatory proteins in the signaling-cascade en route[16] that might prove as potential therapeutic targets in the future.

In this context, the regulation of Toll-like receptor (TLR)signaling in sepsis deserves particular attention. TLR signaling through the MyD88 and TIR-domain-containing adapter inducing interferon-β (TRIF)pathways is crucial for induction of hyperinflammatory responses and tissue injury during sepsis. According to a recent hypothesis, full activation of multiple TLR signaling pathways may lead to hyperinflammation, resulting in organ failure and death, while a moderate signal strength might exert protective functions.[16] Because there is a considerable degree of redundancy between MyD88- and TRIF-dependent signaling pathways in mediating these responses, individual TLR signaling pathways might be targeted by affecting the function of adapter molecules. Hitherto, several negative regulators influencing TLR signaling at different steps of the cascade through competitive inhibition, sequestration or degradation of signaling proteins are known. Radioprotective 105 (RP105) interferes with TLR4 signaling by the inhibition of ligand binding.[17] Single immunoglobulin IL-1-receptor-related molecule (SIGIRR) inhibits LPS-induced signaling by interference with the TIR domain of TLR4 and TLR9.[18] The E3-ubiquitin ligase TLR-ubiquitinating enzyme 3A (Triad3A) inhibits TLR signaling by the promotion of proteolytic degradation of TLR4 and TLR9.[19] ST2-ligand (ST2L) inhibits TLR4 and IL1-R signaling, probably by sequestring MyD88 and Mal.[20] A splice variant of MyD88, MyD88s acts as a dominant negative inhibitor of the MyD88/IRAK complex by preventing phosphorylation of IRAK-1.[21] IRF-4 interacts with the IRAK-1/MyD88/TRAF-6 complex and selectively inhibits IRF-5-dependent TLR signaling.[22,23] In humans, the TRIF pathway is negatively regulated by the TIR adapter sterile α HEAT-Armadillo motif protein (SARM).[24] Another negative regulator of the TRIF pathway is the Src homology 2 (SH2)-domain-containing inositol-5-phosphatase (SHIP)-2, which interferes with the TANK-binding kinase 1 (TBK)1-induced signaling.[25] The function of the IRAK-family members is also controlled by several mechanisms. IRAK-M inhibits the dissociation of IRAK-1 und IRAK-4 from the TLR/MyD88/IRAK signaling complex.[26] Toll-interacting protein (Tollip) inhibits the phosphorylation and kinase activity of IRAK-1.[27] Smad6 abrogates TLR signaling by complex formation with Pelle-interacting protein (Pellino), IRAK-1 and TRAF-6.[28] A20 removes K63-linked ubiquitin residues from TRAF-6 and therefore inhibits TRAF-6-dependent NF-kB activation, while β-arrestin complexes with TRAF6 and averts auto-ubiquitination and NF-kB-activation by TRAF6.

Up to now, little is known about the expression of these negative regulators of TLR signaling in human sepsis and its potential prognostic and eventual therapeutic implications. Their potential of affecting the function of adapter molecules, however, makes them interesting targets for further studies.
